# Hyperkeratosis Lenticularis Perstans (Flegel’s Disease) in a Middle-Aged Man: A Rare Keratinization Disorder

**DOI:** 10.7759/cureus.97966

**Published:** 2025-11-27

**Authors:** Khuzama M Alaseeri, Renad S Alshaikh, Deemah H Attar, Abdulrahman A Alharbi, Khalid Al Hawsawi

**Affiliations:** 1 College of Medicine, King Saud Bin Abdulaziz University for Health Sciences, Jeddah, SAU; 2 Department of Medicine, Ministry of National Guard Health Affairs, Jeddah, SAU; 3 Department of Dermatology, Ministry of National Guard Health Affairs, Jeddah, SAU

**Keywords:** flegel disease, hlp, hyperkeratosis lenticularis perstans, keratinization disorder, skin disease

## Abstract

Flegel disease, also known as hyperkeratosis lenticularis perstans, is a rare cutaneous disease. Here, we report a case of a 56-year-old man who presented with a history of asymptomatic skin lesions that had gradually increased in number over the past year, in the absence of any significant medical history or known predisposing factors for Flegel disease. Skin examination revealed multiple punctate, scaly, blanchable, erythematous papules measuring 1-2 mm in diameter and distributed symmetrically on both lower extremities. Scales were adherent to the peripheral edges of the papules. Palms, soles, scalp, and mucous membranes were not involved, and there was no family history of similar lesions. Punch biopsy revealed epidermal atrophy with an overlying zone of ortho-hyperkeratosis, contrasting with the normal keratin layer. The papillary dermis showed a lichenoid infiltrate and dilated blood vessels. These clinico-pathological findings correlated well with the clinical appearance of multiple scaly erythematous papules and confirmed the diagnosis of Flegel disease. Topical therapy with 0.1% betamethasone valerate ointment was started twice daily. The patient showed mild improvement with reduced scaling and erythema, and no adverse effects were observed. Regular periodic follow-up was scheduled, but the patient was lost to follow-up.

## Introduction

Flegel disease, also known as hyperkeratosis lenticularis perstans (HLP), is a rare and often overlooked skin disorder [1]. It typically affects adults and presents as numerous, small, reddish-brown, scaly papules measuring a few millimeters in diameter, most commonly on the lower legs. Lesions involving the palms, soles, and mucous membranes have been reported. The scales are typically attached to the peripheral margins of the papules. These papules are usually asymptomatic and evolve slowly, and patients often live with this condition for months or even years before seeking medical attention [2,3]. While most reported cases are sporadic, some familial cases have been documented [4].

Emerging evidence indicates that mutations in the SPTLC1 gene, responsible for sphingolipid biosynthesis, contribute to the disease’s pathogenesis by disrupting epidermal lipid metabolism and barrier function, and impairing keratinization [5,6]. These molecular disruptions are thought to impair normal epidermal differentiation, leading to the formation of the characteristic small, scaly papules observed in Flegel disease. Topical therapies, including corticosteroids, 5-fluorouracil, calcipotriene or retinoids, oral retinoids, and psoralen plus ultraviolet A therapy (PUVA), have shown variable results [7]. In many cases, management is primarily directed toward cosmetic outcomes.

In this report, we present a case of a 56-year-old man who developed multiple scaly erythematous papules on the lower legs, confirmed histopathologically as HLP.

## Case presentation

A 56-year-old man with a known history of hypertension on valsartan presented with a history of asymptomatic skin lesions that were slowly increasing in number over the past year. The patient had no significant past medical history and was not on any chronic medications. He denied previous dermatologic conditions, chronic inflammatory diseases, or systemic illnesses. There was no history of recent drug initiation that could provoke lichenoid or keratinization disorders, and no occupational or environmental exposures associated with hyperkeratotic dermatoses. Additionally, there was no family history of similar skin lesions, reducing the likelihood of hereditary variants of Flegel disease. 

**Figure 1:**
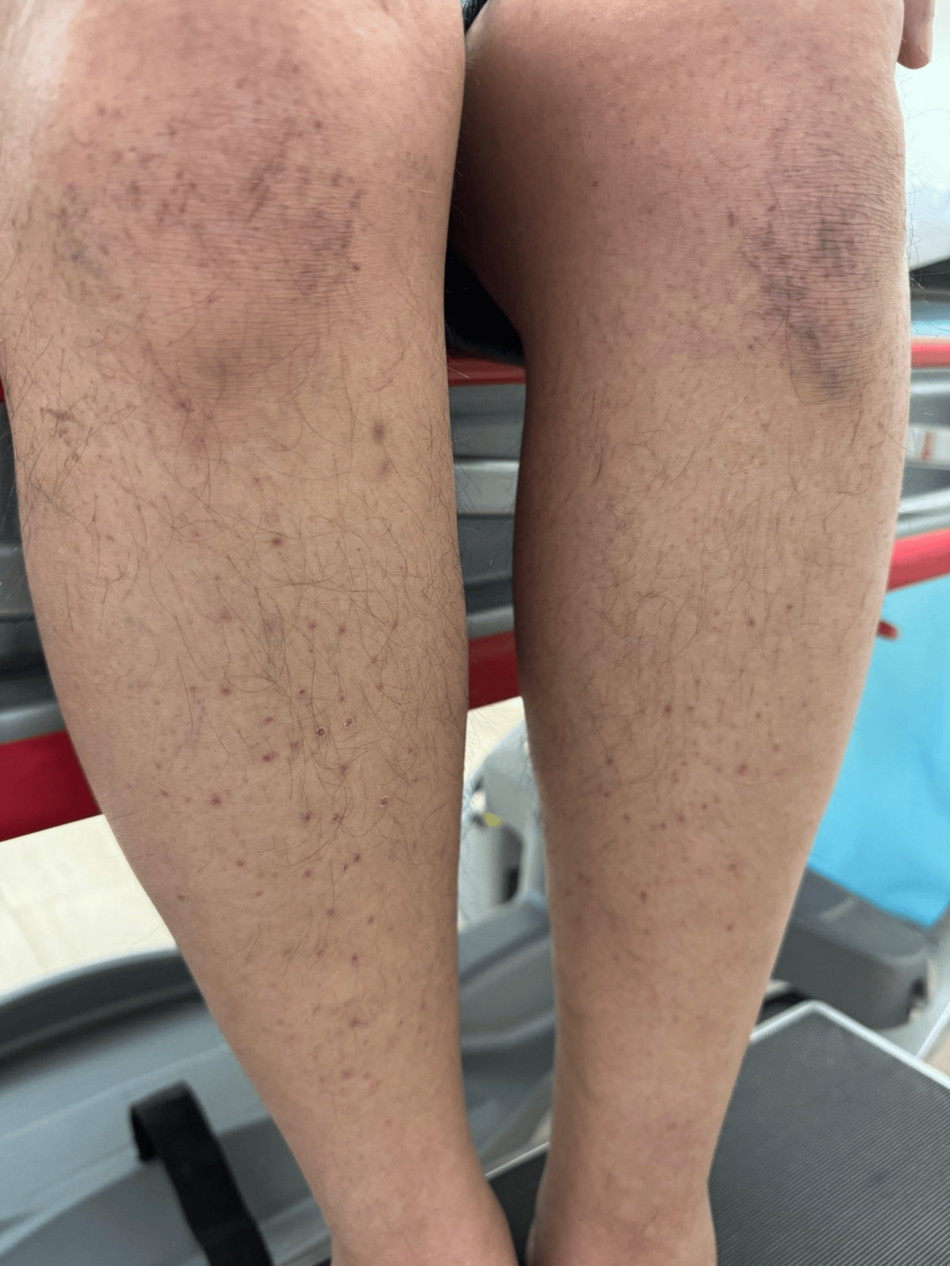
Multiple, discrete, punctate erythematous to brown scaly papules symmetrically distributed over the lower extremities. The scales are adherent to the peripheral edges of the papules.

On clinical examination, multiple punctate, scaly, blanchable, erythematous papules measuring 1-2 mm in diameter were symmetrically distributed over both lower extremities. Scales were adherent to the peripheral edges of the papules (Figure 1). The palms, soles, scalp, and mucous membranes were not involved. Differential diagnoses included lichen nitidus, lichen planus, pigmented purpuric dermatosis, scurvy, porokeratosis, and hyperkeratosis lenticularis perstans (Flegel's disease).

**Figure 2:**
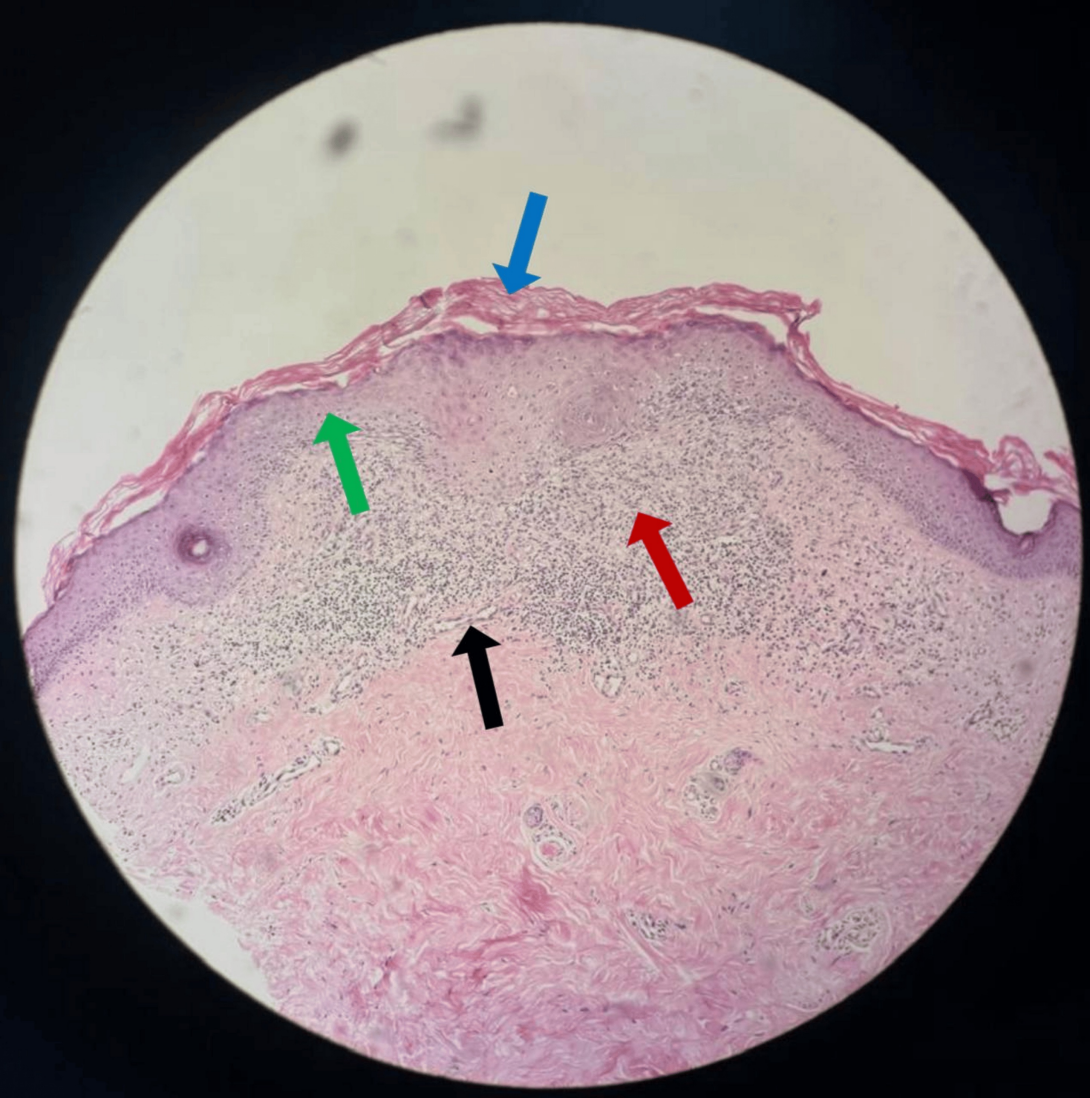
Histopathology showing epidermal atrophy with overlying zone of ortho-hyperkeratosis, contrasting with the normal keratin layer. Papillary dermis showing lichenoid mono-nuclear cellular infiltrate and dilated blood vessels Dark blue arrow: ortho-hyperkeratosis, Green arrow: epidermal atrophy, Red arrow: lichenoid infiltrate, Black arrow: dilated veins

Punch biopsy was obtained. It revealed epidermal atrophy with an overlying zone of ortho-hyperkeratosis, contrasting with the normal keratin layer (Figure 2). The papillary dermis showed a lichenoid infiltrate and dilated blood vessels.

These clinico-pathological findings established the diagnosis of Flegel disease. Topical therapy with 0.1% betamethasone valerate ointment was started twice daily, and periodic follow-up was arranged for the patient. However, the patient did not return for the scheduled follow-up visits, and therefore, the clinical response to topical corticosteroid therapy could not be assessed. 

## Discussion

Flegel disease, or HLP, remains a rare and largely idiopathic keratinization disorder. However, recent genetic findings have begun to clarify its pathogenesis. Jägle et al. identified heterozygous frameshift and splice variants in the *SPTLC1* gene, which encodes the serine palmitoyltransferase-1 enzyme responsible for catalyzing the first step of sphingolipid synthesis (notably ceramides) [4]. These variants trigger nonsense-mediated mRNA decay, markedly reducing SPTLC1 protein levels. Because ceramides and other sphingolipids are essential for normal epidermal differentiation and barrier integrity, SPTLC1 deficiency likely disrupts keratinocyte maturation. This disturbance manifests histologically as compact orthohyperkeratosis with epidermal thinning, the hallmark feature of Flegel disease [4,5]. Impaired sphingolipid synthesis disrupts the normal epidermal lipid barrier, leading to altered keratinocyte differentiation and abnormal keratin accumulation. This disruption contributes directly to the formation of compact orthohyperkeratosis and parakeratosis seen in Flegel disease, providing a mechanistic link between SPTLC1 dysfunction and clinical lesion development [4,5]. 

Expanding on these molecular findings, *SPTLC1 *variants that produce frameshifts or abnormal splicing abolish normal enzyme production [4]. Serine palmitoyltransferase-1 catalyzes the condensation of serine and palmitoyl-CoA to form 3-ketosphinganine, the first committed step in sphingolipid biosynthesis. Loss of this enzymatic activity depletes downstream sphingolipids in the epidermis, compromising the stratum corneum lipid lamellae and overall barrier integrity. Although the precise downstream cascade linking ceramide deficiency to lesion formation remains under investigation, impaired sphingolipid metabolism is thought to dysregulate keratinocyte differentiation, resulting in compact orthokeratosis and epidermal thinning. Thus, the sphingolipid pathway may represent a potential therapeutic target for Flegel disease [4,5].

In addition to genetic mechanisms, environmental and lifestyle factors may modulate disease expression. While no definitive triggers have been established, parallels with other keratinization disorders suggest that viral infections, exogenous agents, or ultraviolet exposure may play contributory roles. Some reports have described associations with endocrine abnormalities, internal malignancies, or postinfectious flares, implying that metabolic or immune disturbances could precipitate lesions, although evidence remains limited [6]. In our patient, no environmental, infectious, or metabolic triggers were identified. He had no history of significant sun exposure, systemic illness, or new medications aside from valsartan for hypertension. Although angiotensin-receptor blockers have been associated with lichenoid drug eruptions, both clinical morphology and histopathology in our case were incompatible with that diagnosis. Moreover, there was no temporal relationship between valsartan initiation and lesion onset. Therefore, a drug-induced etiology was considered unlikely. In the absence of external or metabolic factors, a de novo *SPTLC1* variant remains the most plausible explanation for this presentation.

Differential diagnosis

The presentation of punctate hyperkeratotic papules on the lower limbs required consideration of several differential diagnoses. Lichen planus typically manifests as pruritic, polygonal, violaceous papules with Wickham’s striae, whereas our patient’s lesions were asymptomatic, and histology lacked the characteristic sawtooth rete ridges and dense band-like infiltrate. Lichen nitidus was excluded due to the absence of the ball-and-claw rete pattern or histiocytic granulomas. Pigmented purpuric dermatosis presents with cayenne-pepper-like petechiae and hemosiderin deposition, not true adherent scale, inconsistent with our findings. Scurvy (vitamin C deficiency) was ruled out by normal laboratory results, absence of systemic bleeding, and lack of perifollicular hemorrhages or corkscrew hairs. Porokeratosis (disseminated superficial type) typically shows cornoid lamellae on histology, which were absent in our specimen. Ultimately, the combination of compact orthokeratosis, epidermal thinning, and a lichenoid lymphocytic infiltrate with dilated capillaries confirmed the diagnosis of HLP. This pattern of exclusions, combined with the presence of compact orthohyperkeratosis, epidermal thinning, and a lichenoid infiltrate, aligns with the established clinicopathologic criteria for Flegel disease [4,5].

Management and follow-up

There is currently no curative therapy for Flegel disease, and management remains primarily cosmetic [5,7]. Our patient was treated with 0.1% betamethasone valerate ointment twice daily, achieving only mild softening of papules after three months. This modest improvement aligns with previous reports indicating that available treatments-including topical corticosteroids, 5-fluorouracil, retinoids, vitamin D analogues, phototherapy, and ablative procedures-typically provide only partial and temporary improvement [7]. In our case, treatment outcomes could not be evaluated because the patient was lost to follow-up after the initial visit. Regular clinical monitoring is recommended in patients with Flegel disease to assess lesion stability, evaluate treatment tolerance, and detect any atypical changes early.

Patients should be informed that therapy aims to reduce hyperkeratosis and erythema for cosmetic comfort rather than complete lesion clearance. As Stabile et al. noted, interventions “offer only partial therapeutic responses and are aimed at ameliorating the aesthetic impact of the lesions” [6]. Given the benign and indolent nature of the disease, our management plan emphasizes conservative follow-up every three to six months to monitor lesion evolution and treatment tolerance. Although malignant transformation has not been reported in Flegel disease, periodic surveillance ensures early recognition of any atypical developments.

## Conclusions

This case highlights the importance of considering Flegel’s disease in patients with chronic, asymptomatic hyperkeratotic papules on the legs. Although rare, HLP has distinctive clinicopathologic features; accurate diagnosis relies on correlating the clinical presentation with the biopsy findings. By sharing this example, we aim to raise awareness of this uncommon dermatosis and its hallmark features among clinicians, thereby facilitating timely recognition and appropriate management. Future studies exploring the genetic and molecular mechanisms of keratinization may enhance understanding of Flegel disease and lead to more targeted therapeutic strategies. Incorporating such insights could improve diagnostic precision and long-term management of this rare disorder.
